# Three-Dimensional Facial Imaging: A Comparative Assessment of the Clinical Applicability of State-of-the-Art Technologies for Three-Dimensional Facial Imaging

**DOI:** 10.1155/ijod/8822293

**Published:** 2025-05-14

**Authors:** Thanatchaporn Jindanil, Ranida Ponbuddhichai, Céline Massant, Lianyi Xu, Rocharles Cavalcante Fontenele, Maria Cadenas de Llano-Pérula, Reinhilde Jacobs

**Affiliations:** ^1^Department of Imaging and Pathology, Catholic University of Leuven, Leuven, Belgium; ^2^Department of Oral and Maxillofacial Surgery, University Hospitals Leuven, Leuven, Belgium; ^3^Department of Radiology, Chulalongkorn University, Bangkok, Thailand; ^4^Department of Stomatology, Huazhong University of Science and Technology, Wuhan, China; ^5^Department of Stomatology, Public Health and Forensic Dentistry, University of São Paulo, São Paulo, Brazil; ^6^Department of Oral Health Sciences - Orthodontics, Catholic University of Leuven, Leuven, Belgium; ^7^Department of Dental Medicine, Karolinska Institute, Stockholm, Sweden

**Keywords:** facial scan, similarity, stereophotogrammetry, structured light, three-dimensional imaging, visual perception

## Abstract

**Objective:** To compare the clinical applicability in terms of observer perception, patient perception, and clinical usability of stereophotogrammetry (SPG) and both static and portable structured light (SL) three-dimensional (3D) face scanners. This comparison was based on the perception of medical observers, nonmedical observers, and patients themselves, using two-dimensional (2D) photographs as clinical reference.

**Material and Methods:** Facial images of 20 patients (12 females and eight males) were obtained using a professional camera (clinical reference) and three facial scanners: Vectra H1 (SPG), RAYFace RFS200 (static SL), and iReal 2E (portable SL). Instant similarity rank (ISR) and similarity score (SS) were evaluated by seven medical and six nonmedical observers, and intra- and interobserver reliability were calculated. Patients rated the overall SS (OSS) and comfort. Scanning time, processing time, need for image retake, and user-friendliness were rated by two operators who captured the images.

**Results:** SPG obtained the best ISR, followed by static and portable SL. All scanners showed overall good SS and OSS. Static SL was the fastest, whereas SPG and portable SL recorded same total time. Retake rates for SPG, static SL, and portable SL were 10%, 15%, and 35%, respectively. User-friendliness and comfort ranged from moderate to good for all scanners.

**Conclusion:** All tested scanners show a good clinical applicability, even though each scanner came with specific advantages and drawbacks for clinical use. SPG excelled in instant similarity, but had slower processing times. Static SL offered a balance of speed, comfort, and user-friendliness, though not always the best in similarity. Portable SL had higher retake rates and moderate comfort and user-friendliness. Similarity perception across scanners was comparable for both medical and non-medical observers, highlighting the need for clinicians to consider all scanner features to best meet clinical requirements.

## 1. Introduction

Facial morphology highlights individual uniqueness and plays a crucial role across disciplines as varied as medicine, social sciences, and art [1]. In clinical settings, analyzing facial morphology provides critical information for preoperative diagnosis, treatment planning, postoperative follow-up, and evaluation of the treatment outcome [2–6]. Traditional facial analysis involves two-dimensional (2D) photography or cephalometry on lateral radiographs, where anatomical landmarks are used to perform linear and angular measurements. However, these methods are time-consuming, dependent on precise landmark positioning [7–9], and cannot measure facial volume or soft tissue asymmetry.

The development of three-dimensional (3D) photography and facial scanning has advanced significantly over the years. A key benefit of these technologies is their noninvasive and radiation-free nature [10]. 3D laser scanning, which calculates distances using the triangulation of laser reflections, has become an integral part of clinical practice [11]. However, despite its high-resolution, this method is time-consuming and susceptible to motion artifacts [6, 11–14]. To overcome these issues, newer scanners employ faster techniques such as photogrammetry (PG), stereophotogrammetry (SPG), and structured light (SL) [15–17]. PG involves merging photographs together, while SPG, the current clinical standard, uses multiple images to compute 3D coordinates in postprocessing software [18–20]. Although quicker and more cost-effective than laser scanning, SPG requires extensive postprocessing and can lead to surface inconsistencies [11, 21]. SL technology, which projects light patterns onto surfaces to capture shapes, offers both speed and affordability but can be affected by lighting conditions, leading to motion artifacts and inconsistent results [16, 22–24].

The accuracy of commercial scanners is crucial for clinical applications. Deviations of less than 2 mm are suitable for visual assessments of soft tissue and typically imperceptible to the human eye [16, 25, 26], with discrepancies under 1 mm considered clinically acceptable. Precision and repeatability are critical for clinical use [10], but understanding the patient's perception of the scans is equally important. Therefore, considering the patient's perspective is essential when adopting new technologies in clinical practice. Although studies focusing on the quantitative comparison of different commercial facial scanners, including their differentiation from scans obtained from smartphone applications, there remains a lack of studies focusing on the clinical applicability aspect.

The present study aims to compare the clinical applicability in terms of observer perception (similarity to the real patient), patient perception (similarity and comfort), and clinical usability (time, user-friendliness, and need for image retake) of SPG, static, and portable SL 3D face scanning. This comparison was conducted through the perspectives of medical observers, nonmedical observers, and the patients themselves, using 2D photographs as clinical reference.

## 2. Materials and Methods

### 2.1. Ethical Approval and Sample

This study was conducted in accordance with the World Medical Association's Declaration of Helsinki on Medical Research and was approved by the local Medical Ethics Committee under protocol number S67723. Facial images of 20 patients (12 females and eight males, mean age: 32 ± 6 years) were acquired after obtaining their written informed consent. Patients were required to be adults, capable of remaining still during scanning, and without maxillofacial deformities.

### 2.2. 2D Photography Acquisition

2D photographs were captured in a clinical setting using a professional camera (Nikon D800; Nikon Corporation, Tokyo, Japan). The camera was mounted on a tripod and positioned at a fixed distance from a chair to standardize the image capturing process. Five clinical photos were captured for each patient in nonsmiling and relaxed positions (frontal, left oblique, right oblique, and lateral). These images were subsequently exported in Joint Photographic Experts Group (JPEG) format to serve as the “real patient reference” during the questionnaire session.

### 2.3. 3D Photography Acquisition

The same patients were scanned three times in nonsmiling and resting positions using three scanners: portable SPG, static SL, and portable SL, following the manufacturers' protocols with operators receiving calibration prior to the study.

For 3D SPG images, the Vectra H1 (Canfield Scientific Inc., Parsippany, NJ, USA) was utilized following calibration. This 3D camera utilizes a stereooptic system equipped with ranging lights to aid in patient positioning, accompanied by a modular intelligent flash unit. The camera offers images with a geometric resolution of 0.8 mm and a capture volume of 165 mm × 270 mm × 100 mm (*x*, *y*, *z*) with a scan time of 2.0 ms [27]. During each scan, patients were instructed to remain still. The first image capture involved positioning the camera 30 cm below the midface at chest level, angling it upwards at a 45° angle towards the patient's right side. The second image was taken from the front, aligning the camera at the patient's nose level and directly facing them. The third image, capturing the left side, was taken using the same approach as the right side. These three images were then stitched together into a 3D image using VECTRA Face Sculpture software and exported in Object File Format (OBJ).

3D SL scans were obtained using the RAYFace RFS200 (RAY Co., Ltd, Gyeonggi-do, South Korea), a static blue–white SL machine, following calibration. This machine is equipped with six cameras (left, right, bottom, teeth left, teeth right, and face/frontal), along with three LED lights to illuminate the patient from the left, right, and bottom angles. It offers a scan area of 220 mm × 300 mm and a resolution of 1440 × 1080 pixels, producing 24-bit true color images with a white balance of 5600–5800 K under normal LED lighting [28]. The scans were reconstructed using RAYFace software and subsequently exported in OBJ format.

Finally, portable SL scans were captured using the iReal 2E (Scantech, Hangzhou, China), a portable infrared vertical-cavity surface-emitting laser diode (VCSEL) SL machine, following calibration. This device is equipped with a central VCSEL and three sets of supplementary invisible light sources, a camera group, and auxiliary lights. It offers a scanning area of up to 580 mm × 550 mm, achieves an accuracy of up to 0.1 mm, and operates at a maximum scanning rate of 1,500,000 points per second [29]. The captured scans were processed and reconstructed using iReal software and subsequently exported in OBJ format.

**Figure 1:**
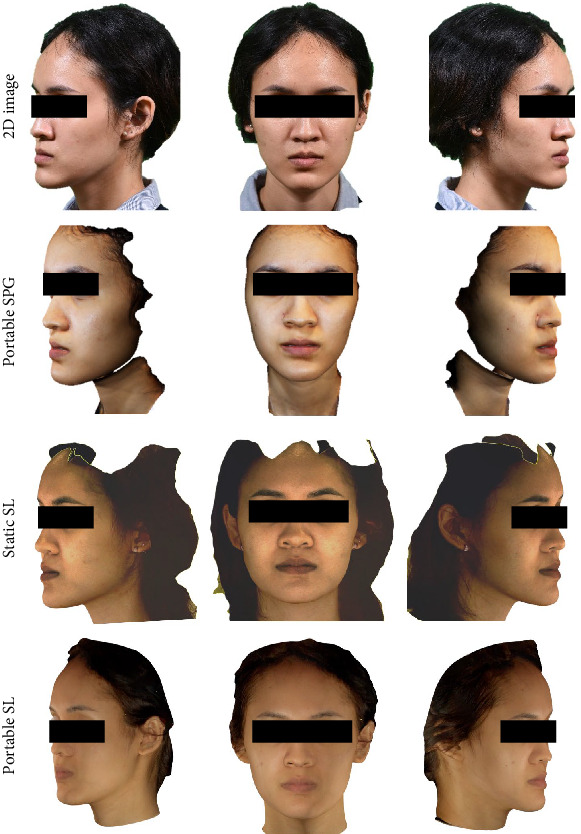
Example of images from each acquisition from different scanner based on different technology. 2D image: two-dimensional image (clinical standard), SL, structured light; SPG, stereophotogrammetry.

Figure 1 illustrates examples of images from both 2D and 3D photography acquisitions. All OBJ files were subsequently processed using Blender (version 3.2, Blender Foundation, Amsterdam, Netherlands) to align and adjust the dimensions and angles of the scans. This ensured that each scan was perpendicular to the axis and uniformly angled.

### 2.4. Qualitative Assessment of 3D Photography Variants Using 2D as Clinical Reference: Similarity

To qualitatively assess observer perception, 13 observers were involved: seven medical observers (including three fifth-year medical students, a general dentist, an oral and maxillofacial radiologist, a prosthodontist, and a paedodontist) and six nonmedical observers (comprising two engineers and four biomedical scientists). They were initially trained to reviewing 3D images and discussing the definitions of “similarity” and “dissimilarity” to the real patient. Subsequently, they were calibrated by scoring three practice patients (i.e., not part of the study sample) before starting the formal scoring session. During the evaluation, each observer assessed the similarity to the real patient of SPG, static SL, and portable SL 3D face scans. For reference, 2D images of each patient were displayed alongside the corresponding 3D scans, which were rotated 180° horizontally to evaluate both frontal and lateral profiles.

Similarity assessment began with an instant similarity rank (ISR), where observers ranked the 3D scans (SPG, static, and portable SL) of each patient on a scale of 1–3, with 1 indicating the best image and 3 the worst. Additionally, the similarity score (SS) index was employed based on the criteria outlined in Table 1. Observers evaluated each scan according to criteria for upper (trichion–nasion), middle (nasion–subnasale), and lower (subnasal–menton) facial thirds. Each criterion (completeness of data, light conditions, skin texture, and motion artifacts) allowed deductions of up to five points in total. Observers could deduct only one point per subcriterion within each criterion. The highest possible SS was 5, indicating the best match, while the lowest was 1. Three SS were calculated for each scan based on evaluations from the three facial regions.

### 2.5. Qualitative Assessment of 3D Photography Variants Using 2D as Clinical Reference: Patient Perception

In addition, to broadly evaluate patient perception, 20 patients were asked to rate the similarity of the SPG, static SL, and portable SL 3D images with themselves immediately after the scans. They used a five-point Likert scale (1: poor, 2: fair, 3: moderate, 4: good, and 5: very good), which contributed to an overall SS (OSS). Furthermore, these 20 patients were asked to rate the comfort of the entire scanning process on a scale from 1 to 3 (1: poor, 2: moderate, and 3: good).

### 2.6. Qualitative Assessment of 3D Photography Variants Using 2D as Clinical Reference: Clinical Usability

Clinical usability was evaluated across five variables in this study. Scanning time, which refers to the duration the machine operated during scanning, was measured to gauge efficiency. Processing time encompassed the reconstruction process within the software, while total time represented the combined duration of scanning and processing. Need for image retake was calculated as a percentage of total images requiring re-scanning per session, providing insight into reliability. User-friendliness, crucial for operational ease, was assessed by operators (TJ, RP) using a scale ranging from 1 (poor) to 3 (good).

### 2.7. Statistical Analysis

Two one-sided *t*-tests (TOST) procedure with a predefined equivalence margin of ±1, the 90% confidence interval for the mean difference (–0.815, 0.967) was entirely within the margin, supporting statistical equivalence (*α* = 0.05).

To assess the reliability of the medical and nonmedical observers, the ratings of six observers (three medical and three nonmedical observers) were repeated over an 8-week interval. Intra- and interobserver reliability were evaluated using Fleiss Kappa analysis, where the percentage of concordance was calculated. The agreement results were interpreted according to the Landis and Koch scale: 0–0.2: slight; 0.2–0.4: fair; 0.4–0.6: moderate; 0.6–0.8: substantial; 0.8–1.00: almost perfect [29].

For the similarity assessment, both SS and OSS were initially reclassified into a three-level scale: scores 1–2 were categorized as 1 (poor), 3 as 2 (moderate), and 4–5 as 1 (good). Afterwards, SS and OSS were descriptively analyzed using the mode, given they are categorical variables. The SS provided by medical and nonmedical observers for the three 3D scanners were compared using the Friedman test. The OSS provided by the subjects regarding their own scans were compared using Wilcoxon pairwise comparison. The ISR was expressed as a percentage to indicate how many observers ranked the similarity of each scanner as 1 (best), 2, or 3 (worst).

For clinical usability, the scanning time, processing time, and total time of the three 3D scanners were first descriptively reported with means and standard deviations. These results were then compared using analysis of variance (ANOVA). Additionally, the need for image retakes was presented as percentages, comfort ratings were described using modes and user-friendliness ratings were reported with ranges. All statistical analysis were conducted using SPSS version 29.0 (IBM Corp, New York, USA) with a significance level of 0.05.

The study was preregistered in Open Science Framework (https://doi.org/10.17605/OSF.IO/2ZF3A).

## 3. Results

### 3.1. Reliability Assessment

The intraobserver reliability ranged from 0.71–0.88, while interobserver reliability was 0.66–0.98 (for medical observers) and 0.69–0.86 (for nonmedical observers), respectively, indicating substantial to almost perfect reliability.

### 3.2. Qualitative Assessment of the 3D Scans: Similarity

**Figure 2:**
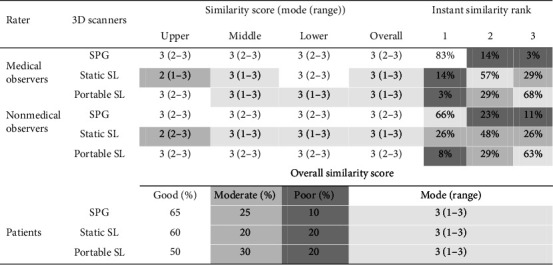
Similarity perception ratings by patients, medical, and nonmedical observers across facial scanners. SL, structured light; SPG, stereophotogrammetry. Similarity score: Similarity scoring was based on data completeness, light conditions, skin texture, and motion artefacts, compared to 2D images rated by observers (1: poor similarity, 2: moderate similarity, and 3: good similarity). Instant similarity rank reflects observer preferences for 3D scans (1: the best, 2: fair, and 3: the worst). Overall similarity score represents the similarity level between the patients themselves immediately after the scans was obtained rated by patients (1: poor similarity, 2: moderate similarity, and 3: good similarity). Color interpretation: no highlight/white: good, pale gray: fairly good, light gray: moderate, and dark gray: poor.

Figure 2 presents results for SS, ISR, and OSS per scanner, assessed by patients, medical observers, and nonmedical observers. According to ISR, 83% of medical observers rated SPG as the best option, followed by static (14%) and portable SL (3%). Nonmedical observers confirmed this ranking, although percentages differed: 66% favored SPG, while static and portable SL received 26% and 8%, respectively, indicating higher preference for static and portable SL compared to medical observers. The overall mode of the SS across all three scanners was consistently 3 (good).

Table 2 displays the statistical comparison of the SS per scanner by medical and nonmedical observers. SPG was rated significantly more similar to the real patient than static SL in the upper and middle facial thirds, according to both medical and nonmedical observers (*p*  < 0.01). In contrast to ISR results, portable SL was rated significantly more similar to the patient than static SL in the upper facial third by both groups of observers (*p*  < 0.01). Nonmedical observers also found SPG significantly more similar than portable SL in the middle facial third (*p*=0.013). However, there was no statistically significant difference among the devices tested for the lower facial third (*p*  > 0.05).

### 3.3. Qualitative Assessment of 3D Photography Variants Using 2D as Clinical Reference: Patient Perception

Patients did not perceive significant differences among the devices tested; overall, the SS rated by subjects across all three scanners was consistently within category 3 (Figure 2). Patients found SPG to have the highest similarity with the real patient (66%), closely followed by static SL (60%), and portable SL (50%). In terms of comfort, patients rated static SL as providing good comfort, while SPG and portable SL were perceived as offering moderate comfort.

### 3.4. Qualitative Assessment of the 3D Scans: Clinical Usability

**Figure 3:**
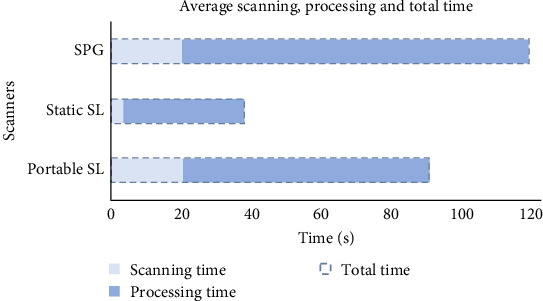
Average scanning, processing, and total time of portable stereophotogrammetric (SPG) scanner, static structured light (SL) scanner, and portable SL scanner.

**Figure 4:**
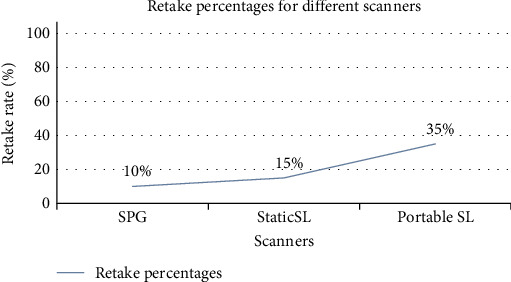
Retake percentages of portable stereophotogrammetric (SPG) scanner, static structured light (SL) scanner, and portable SL scanner.

Static SL was the fastest in terms of scanning, processing, and total time (*p*  < 0.001), all statistically significant (*p*  < 0.001), as shown in Figure 3. There was no significant difference in scanning time between SPG and portable SL; however, SPG had a slower processing time (*p*  < 0.001).  Figure 4 presents the retake rates for SPG, static SL, and portable SL, recorded at 10%, 15%, and 35%, respectively. Regarding user-friendliness, static SL was rated as good, while both SPG and portable SL received a moderate rating from the two operators responsible for capturing the images.

## 4. Discussion

In the digital era, facial scanning has gained significant importance as a fundamental component of the digital workflow. This study highlights that each scanner, employing distinct imaging principles, presents unique strengths and weaknesses concerning similarity and clinical utility. Our subjective analysis revealed that medical and nonmedical observers prioritize different factors when assessing scans. This insight is crucial in selecting the most suitable scanners for clinical implementation, striking a balance between technological capability and practical usability. The inclusion of highly educated nonmedical observers, such as (bio)engineers and biomedical scientists, reflects their frequent involvement in image capture and postprocessing tasks.

The findings on similarity are noteworthy: despite portable and static SL scoring lower in ISR percentages, their SS in specific facial region and OSS were deemed good and comparable to other scanners, suggesting their qualitative adequacy for clinical purposes. Several factors can influence similarity assessments, including lighting conditions, hair, surface color, and texture of the scans. Variations in lighting conditions, influenced by fixed or movable light sources among scanners, coupled with diverse facial morphology, particularly around the orbital region, can affect perceptions of dissimilarities in light conditions and data completeness in the upper facial region [5]. This may explain why most differences were noted in the upper and middle facial thirds. Additionally, prolonged scanning times and natural occurrences of eye blinking can introduce artifacts into the scan [30, 31].

Color, a fundamental signal in basic vision, plays a crucial role in discrimination and holds significant relevance in clinical settings [32, 33]. Due to inherent optical properties, scans obtained from laser and SL scanners typically offer lower color resolution, potentially influencing bias in scanner perception and impacting ISR, SS, and OSS similarity assessments [23]. While geometric properties provide valuable technical insights into scanner capabilities, overlooking color may not fully meet clinical requirements. It is possible that better geometric accuracy may inherently lead to longer scanning and processing times. However, achieving high geometric accuracy is essential in the clinical workflow, intended to be used in detailed procedures, such as 3D printing of surgical guides or presurgical planning, as it forms the foundation for successful 3D printing. As discussed by Salazar-Gamarra et al. [24], minor surface imperfections in the model may be acceptable for the prosthesis if final adjustments are made manually. Also, perfect geometric accuracy might be less important when records are merely used for clinical discussion. Irrespective of this, this study consistently showed no distinct preference for any scanner based on ISR, SS, and OSS, indicating a balanced perception across all scans.

Considering patient perception and clinical usability, the ideal scanner should prioritize patient comfort, affordability, user convenience, and the ability to produce fast yet clinically acceptable scan quality. However, each scanner model comes with its distinct advantages and drawbacks. A faster scanner is particularly beneficial for less cooperative patients, while a slower scanner might capture finer details more effectively with more cooperative subjects. The current results indicated that static SL offered fast scanning, good subject comfort, and operator convenience, making it particularly suitable for children or patients with special needs. However, portable SL and SPG also demonstrated moderate to good comfort and user-friendliness. Despite this, their slower processing time may limit their use in routine practice. SPG is ideal for detailed anatomical reconstruction, such as maxillofacial prostheses, while portable SL stands out for its portability, affordability, and ease of use in various settings with minimal preparation compared to other types of scanners. Possible solutions to mitigate the limitations of each scanner include supplementing the system with powerful computer hardware to enhance software processing, thereby reducing processing time. Texture mapping or improved lighting conditions can also significantly improve texture and color capture. In addition, familiarity and experience with the scanner and its system can positively impact both the overall user experience and the quality of the results. Clinicians must carefully consider all aspects and features of a scanner to strike the right balance that aligns with their clinical requirements.

The primary limitation of this study is its qualitative nature, which precluded gathering quantitative data on scan accuracy, repeatability, and conditions such as patient positioning with varying facial expressions, such as smiling and nonsmiling. A more diverse cohort, including variations in age, gender, ethnicity, and facial characteristics (e.g., patients with maxillofacial deformities), could enhance the clinical relevance of the study. Additionally, the inclusion of highly educated nonmedical observers may not fully represent the perceptions of the general population, as their viewpoints could differ from those of laypersons. Despite these limitations, this research offers valuable insights into aspects such as user-friendliness and comfort that are not easily quantifiable. Furthermore, the current study did not include all available scanning technologies on the market. Last, the limited number of observers involved may limit how broadly applicable the findings are. Future research should involve a broader range of scanners and observers from diverse specialties to thoroughly explore these aspects.

## 5. Conclusion

The tested scanners (SPG, static SL, and portable SL) demonstrated good clinical applicability, even though each scanner came with specific advantages and drawbacks for clinical use. SPG received the highest similarity ratings from both medical and nonmedical observers and required the fewest image retakes, despite slightly slower processing time. Static SL emerged as the best compromise in terms of speed, comfort, and user-friendliness, although it did not consistently achieve the highest SS. Portable SL showed less favorable outcomes with higher retake rates, moderate comfort, and user-friendliness ratings. Importantly, the tested scanners all showed a clinically satisfactory similarity perception as compared to the human reference. However, clinicians might be advised to assess the particular features of each scanner, to ensure whether this aligns with specific clinical needs.

## Figures and Tables

**Table 1:** Criteria to evaluate the similarity of 3D images taken with different scanners as compared to the facial appearance on 2D clinical photographs.

Criteria	Subcriteria	Example	
		Images	Definition
Completeness of data	Data loss in the region of interest, presence of holes, difficulty in tracing the edges of areas such as the hairline.	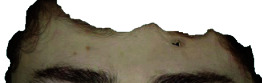	- Data loss in the region of interest (forehead) with the presence of hole

Light condition	Too much or insufficient light, limiting data visualization.	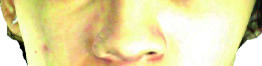 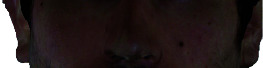	- Too much light - Insufficient light

Skin texture	Patches, poor resolution, or unnatural texture appearance.	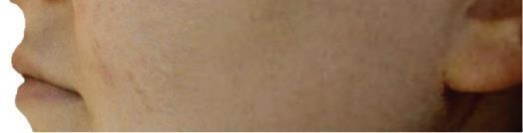	- Patches and unnatural texture appearance at cheek area

Motion artifacts	Insufficient resolution, particularly in the eye and mouth areas such as blurring, eye blinking, mouth twitching, and minor muscle movements.	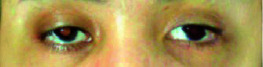 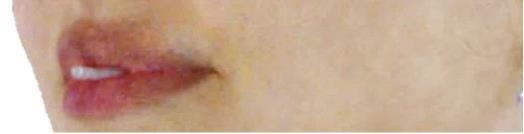	- Double layers of upper eyelids (eye blinking) - Mouth twitching

**Table 2:** Statistical comparison of the similarity scores per scanner given by medical and nonmedical observers according to the Friedman test.

Facial region	Scanners	Medical observers (*p* value)	Non-medical observers (*p* value)
Upper third	SPG–static SL	<0.001*⁣*^*∗*^	<0.001*⁣*^*∗*^
	SPG–portable SL	>0.999	>0.999
	Static SL–portable SL	<0.001*⁣*^*∗*^	<0.001*⁣*^*∗*^

Middle third	SPG–static SL	0.011*⁣*^*∗*^	<0.001*⁣*^*∗*^
	SPG–portable SL	>0.999	0.013*⁣*^*∗*^
	Static SL–portable SL	>0.999	>0.999

Lower third	SPG–static SL	0.991	>0.999
	SPG–portable SL	>0.999	>0.999
	Static SL - Portable SL	>0.999	>0.999

Abbreviations: SL, structured light; SPG, stereophotogrammetry.

*⁣* ^*∗*^ *p*  < 0.05.

## Data Availability

The data that supports the findings of this study are available in the supporting information of this article.
